# Supplementary material for: Adaptation to transients disrupts spatial coherence in binocular rivalry
Source: Sci Rep. 2020 May 26;10:8673. doi: 10.1038/s41598-020-65678-7 (PMC7251118; doi:10.1038/s41598-020-65678-7)
Supplement: Supplementary file 1 — Supplementary information. [file 41598_2020_65678_MOESM1_ESM.pdf]

Supporting information legends and tables

Title

Adaptation to transients disrupts spatial coherence in binocular rivalry

Authors

Marnix Naber, Sjoerd Stuit, Yentl de Kloe, Stefan Van der Stigchel, & Chris L.E. Paffen

Affiliation

Experimental Psychology, Helmholtz Institute, Faculty of Social and Behavioral Sciences, Utrecht University, Heidelberglaan 1, 3584CS Utrecht, The Netherlands

Supplementary Table 1. Post-hoc, paired, two-tailed t-test comparisons of fraction mixed percepts between conditions for Experiment 1.

	No conflict (M = 0.34; SD = 0.18)	Rivalry conflict (M = 0.31; SD = 0.16)	Rivalry-plaid (M = 0.29; SD = 0.15)	Plaid no conflict (M = 0.29; SD = 0.17)
Conflict (M = 0.36; SD = 0.18)	t = 1.870, p = .037	t = 3.291, p = .003	t = 4.350, p < .001	t = 4.443, p < .001
No conflict		t = 1.924, p = .066	t = 4.013, p < .001	t = 4.088, p < .001
Rivalry conflict			t = 2.231, p = .035	t = 1.908, p = .068
Rivalry-plaid				t = -0.140, p = .890

Supplementary Table 2. Post-hoc**,** paired, one-tailed t-test comparisons of fraction mixed percepts between conditions for Experiment 2.

	50% Contrast (M = 0.39; SD = 0.16)	25% Contrast (M = 0.39; SD = 0.17)	No conflict (M = 0.38; SD = 0.15)
Conflict (M = 0.43; SD = 0.17)	t = 2.065, p = .026	t = 1.874, p = .038	t = 2.695, p = .007
50% Contrast		t = 0.433, p = .335	t = 0.821, p = .211
25% Contrast			t = 0.315, p = .378

Supplementary Table 3. Post-hoc**,** paired, one-tailed t-test comparisons of fraction mixed percepts between conditions for Experiment 3.

	100% Rivalry plaid conflict (M = 0.30; SD = 0.14)	50% Rivalry plaid conflict (M = 0.27; SD = 0.15)	Single orientation no conflict (M = 0.29; SD = 0.15)
Conflict (M = 0.37; SD = 0.19)	t = 2.700, p = .007	t = 4.079, p < .001	t = 3.446, p = .001
100% Rivalry-plaid conflict		t = 1.787, p = .045	t = 0.400, p = .347
50% Rivalry-plaid conflict			t = -1.524, p = .072

Supplementary Table 4. Averaged input values for the general linear model predicting the fraction mixed percept per condition (columns) and transient type (rows). The values roughly correspond to the plotted lines in Figure 2c, 3c, and 4c. The monocular contrast values correspond to the fraction change in contrast. The perceptual orientation values correspond to the predefined alternation rates of 0.94Hz, except for the rivalry and rivalry-plaid conditions, which were based on the measured alternation rates of each individual during the test period. For the rivalry-plaid conditions we decreased the parameters with a factor of two as rivalry dynamics slow down when orientations overlap between eyes. The monocular orientation values were similarly based on the rate of 0.94Hz or no changes. The conflict values indicate whether conflict was present (1) or absent (0).

**Transient type**	Conflict	No conflict	Rivalry conflict	Rivalry-plaid conflict	Plaid no conflict	Conflict	50% contrast conflict	25% contrast conflict	No conflict	Conflict	Plaid-rivalry conflict	50% Plaid-rivalry conflict	Single orientation no conflict
Monocular contrast (CO)	1	0	0	0	0	1	.5	.25	0	1	1	.5	1
Perceptual orientation (PO)	.94	.94	.33	.17	0	.94	.94	.94	.94	.94	.17	.08	0
Monocular orientation (MO)	0	.94	.94	0	0	0	0	0	.94	.94	0	.94	.94
Conflict (C)	1	0	1	1	0	1	1	1	0	1	1	1	0
